# Antimicrobial resistant and enteropathogenic bacteria in ‘filth flies’: a cross-sectional study from Nigeria

**DOI:** 10.1038/s41598-020-74112-x

**Published:** 2020-10-12

**Authors:** Francis Chinedu Onwugamba, Alexander Mellmann, Victor Oluoha Nwaugo, Benno Süselbeck, Frieder Schaumburg

**Affiliations:** 1 Institute of Medical Microbiology, University Hospital Münster, Domagkstr. 10, 48149 Münster, Germany; 2 Institute for Hygiene, University Hospital Münster, Robert-Koch-Straße 41, 48149 Münster, Germany; 3 Abia State University, PMB 2000, Uturu, Abia State Nigeria; 4 Center for Information Processing, University of Münster, Röntgenstraße 9-13, 48149 Münster, Germany

**Keywords:** Biogeography, Bacterial infection

## Abstract

‘Filth flies’ facilitate the dispersal of pathogens between animals and humans. The objective was to study the intestinal colonization with antimicrobial resistant and enteropathogenic bacteria in ‘filth flies’ from Nigeria. Flies from Southern Nigeria were screened for extended-spectrum β-lactamase producing *Enterobacterales* (ESBL-E), *Staphylococcus aureus*, *Salmonella* sp., *Shigella* sp., *Campylobacter* sp. and *Yersinia enterocolitica* by culture. ESBL-E were tested for *bla*_SHV_, *bla*_CTX-M_ and *bla*_TEM_; *S. aureus* was screened for enterotoxins. *Spa* typing and multilocus sequence typing (MLST) was done for *S. aureus* and MLST for *Escherichia coli*. Of 2,000 flies, 400 were randomly collected for species identification. The most common species were *Musca domestica* (44.8%, 179/400), *Chrysomya putoria* (21.6%, 85/400) and *Musca sorbens* (18.8%, 75/400). Flies were colonized with *S. aureus* (13.8%, 275/2,000) and ESBL-E (0.8%, 16/2,000). No other enteropathogenic bacteria were detected. The enterotoxin *sei* was most common (26%, 70/275) in *S. aureus*, followed by *sea* (12%, n = 32/275). Four *S. aureus* isolates were methicillin resistant (*mecA* positive, t674 and t5305, ST15). The *bla*_CTX-M_ (n = 16) was the most prevalent ESBL subtype, followed by *bla*_TEM_ (n = 8). ‘Filth flies’ can carry antimicrobial resistant bacteria in Nigeria. Enterotoxin-positive *S. aureus* might be the main reason for food poisoning by ‘filth flies’ in the study area.

## Introduction

Diptera (true flies) are the most abundant and diversified endopterygota (or holometabola) of the insect order, with more than 180 families of about 158,000 described species^1,2^. ‘Filth flies’ (diptera) are universal, ubiquitous, coprophagic and synanthropic (living in close association with humans) insects that breed in garbage, animal and human faeces^3^. Known to serve as vectors of many pathogens, house flies can disperse pathogens through a flight distance of about 7 km between animals and humans^4^.

Flies transmit pathogens through three routes: mechanical translocation from the exoskeleton, regurgitation and defecation^3^. During feeding, flies can either pick up pathogens on its exoskeletal surfaces or ingest fluids contaminated with pathogens. Ingested pathogens can multiply in the crop (a blind sac of the digestive tract in higher flies) and after regurgitation which coined the term of “bioenhanced transmission”^5^.

Antimicrobial resistance (AMR) affects both humans and animals and antimicrobial resistant bacteria can be transmitted between animals and humans in both direction. This challenge is considered in the “one Health” approach. The most widespread resistance mechanisms in *Enterobacterales* is based on plasmid-mediated production of extended spectrum β-lactamases (ESBL) which hydrolyse β-lactam rings, thereby reducing the efficacy of cephalosporins and monobactams^6^. Flies are important reservoirs and vectors of antimicrobial resistant bacteria (such as methicillin resistant *Staphylococcus aureus*, ESBL-producing *Enterobacterales* [ESBL-E])^3,7^. One short report suggests that antimicrobial resistant bacteria can also be detected in flies (n = 25) in sub-Saharan Africa^8^. However, the true burden of AMR in ‘filth flies’ in Africa is unknown and it is currently unclear which fly species are the main vectors of ESBL-E and enteropathogenic bacteria. Therefore, the objective of this study was to analyse the colonization rates of ‘filth flies’ from Southern Nigeria with ESBL-E, *S. aureus* and other enteropathogenic bacteria and to identify those fly species which are mainly colonized with these target organisms.

## Results

### Sampling sites

**Figure 1:**
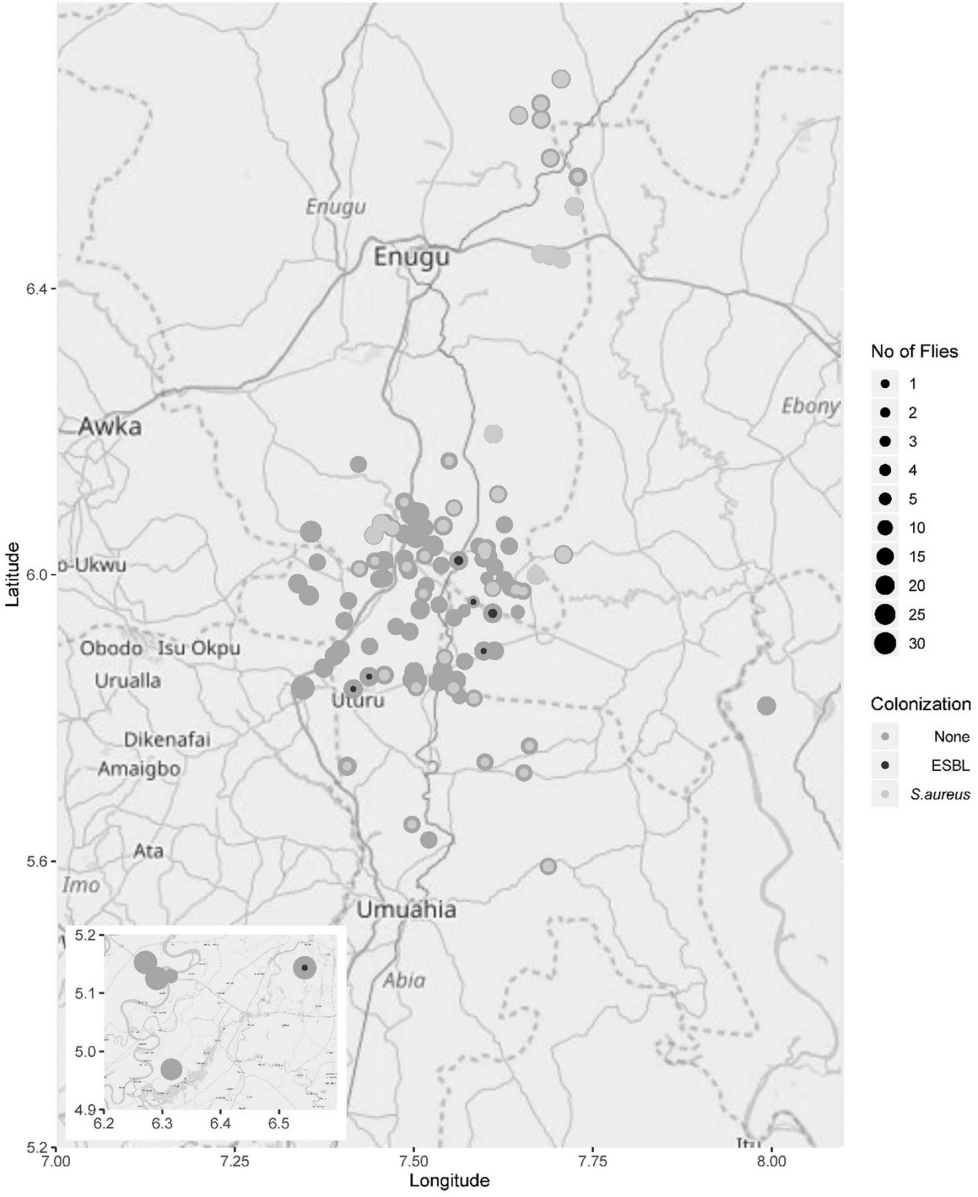
Study area and sampling sites. Each dot represents one of the 109 sampling sites. The size of each dot represents the number of flies. The size of the inner circle corresponds to the number of ESBL-E (black circle) or *S. aureus* colonized flies (light-grey circle) found in that specific site. Grey dots without inner black or light-grey circle indicate that no flies were colonized with ESBL-E or *S. aureus*. Sampling sites were positive for *S. aureus* (n = 45; 41%) or ESBL-E (n = 7; 6%). We used geodata from openstreetmap (https://www.openstreetmap.org) to render our own map using the package “ggplot2” as implemented in “R” (OpenStreetMap contributors)^25^.

In total, 109 sites were sampled, with a total area cover of 15,075,000 m^2^ (Fig. 1). A total of 2,000 flies were captured with a mean number of 18.3 (± SD 5.8) flies per sampling spot. Approximately 40 flies were caught per hour. The mean (± SD) atmospheric parameters were a temperature of 26 °C (± 1.6), relative humidity of 86.5% (± 6.2), windfall of 5.6 Beaufort (± 1.4), air pressure of 1012.4 hPa (± 1.3) and 13 sunshine hours (± 0.4). The sampled sites were urban (8.3%, 9/109), semi-urban (61.4%, 67/109) and rural (30.3%, 33/109). Within a 10 m radius of each sampling site (n = 109), we recorded decomposing organic matter (79.8%, 87/109), refuse dump (84%, 92/109) and animal faeces (49%, 49/109, Table 1).

**Table 1:** Association of *Staphylococcus aureus* in flies with environmental factors.

Environmental factors on day of sampling	Sample sites					
	Detection of *S. aureus* (n = 45),	No detection of *S. aureus* (n = 64)	Crude OR (95% CI)^a^	Crude *p*-value^a^	Adjusted OR (95% CI)^b^	Adjusted *p*-value^b^
Animal faeces [n (%)]	23 (51.1)	26 (40.6)	1.5 (0.7–3.2)	0.28	NA	NA
Refuse dump [n (%)]	37 (82.2)	55 (85.9)	0.8 (0.3–2.1)	0.60	NA	NA
Decomposing organic matter [n (%)]	37 (82.2)	50 (78.1)	1.3 (0.5–3.4)	0.60	NA	NA
Urban [n (%)]	9 (20)	0 (0)	NA	1	NA	1
Semi-urban [n (%)]	15 (33.3)	52 (81.3)	0.2 (0.1–0.4)	< 0.001	0.07 (0.01–0.4)	0.001
Rural [n (%)]	21 (46.7)	12 (18.7)	Reference	NA	NA	NA
Mean temperature [°C (± SD)]	25.5 (0.8)	26.6 (1.9)	0.5 (0.4–0.8)	0.002	0.6 (0.4–1)	0.03
Air pressure [hPa (± SD)]	1012.9 (1.3)	1012.1 (1.2)	1.6 (1.2–2.2)	0.003	1.6 (1.1–2.3)	0.01
Relative humidity [% (± SD)]	88.5 (4.4)	85.1 (6.9)	1.1 (1.0–1.2)	0.007	1.0 (0.9–1.2)	1
Windforce [Bft. (± SD)]	5.4 (1.6)	5.8 (1.3)	0.8 (0.6–1.0)	0.09	0.7 (0.5–1)	0.04
Sunshine hours [h (± SD)]	12.9 (0.3)	12.7 (0.5)	5.6 (1.5–19.9)	0.01	1.4 (0.2–8.6)	0.8

*SD* standard deviation.

^a^From an univariable analysis.

^b^From a multivariable logistic regression analysis with a stepwise backward elimination. All environmental factors potentially associated with the detection of *S. aureus* in flies (*p* < 0.25) were entered in the multivariable model.

### Intestinal culturome of flies

The most common species of the intestinal culturome of 82 randomly selected flies were *Bacillus cereus* (73%, 60/82), followed by *Enterococcus faecalis* (27%, 22/82), *Enterococcus faecium* (24%, 20/82), *Clostridioides tertium* (21%, 17/82), *Bacillus licheniformis* (11%, 9/82), *Bacillus subtilis* (11%, 9/82), *Enterococcus hirae* (11%, 9/82) and others (70%, 57/82, Figure S1).

### *S. aureus*

**Figure 2:**
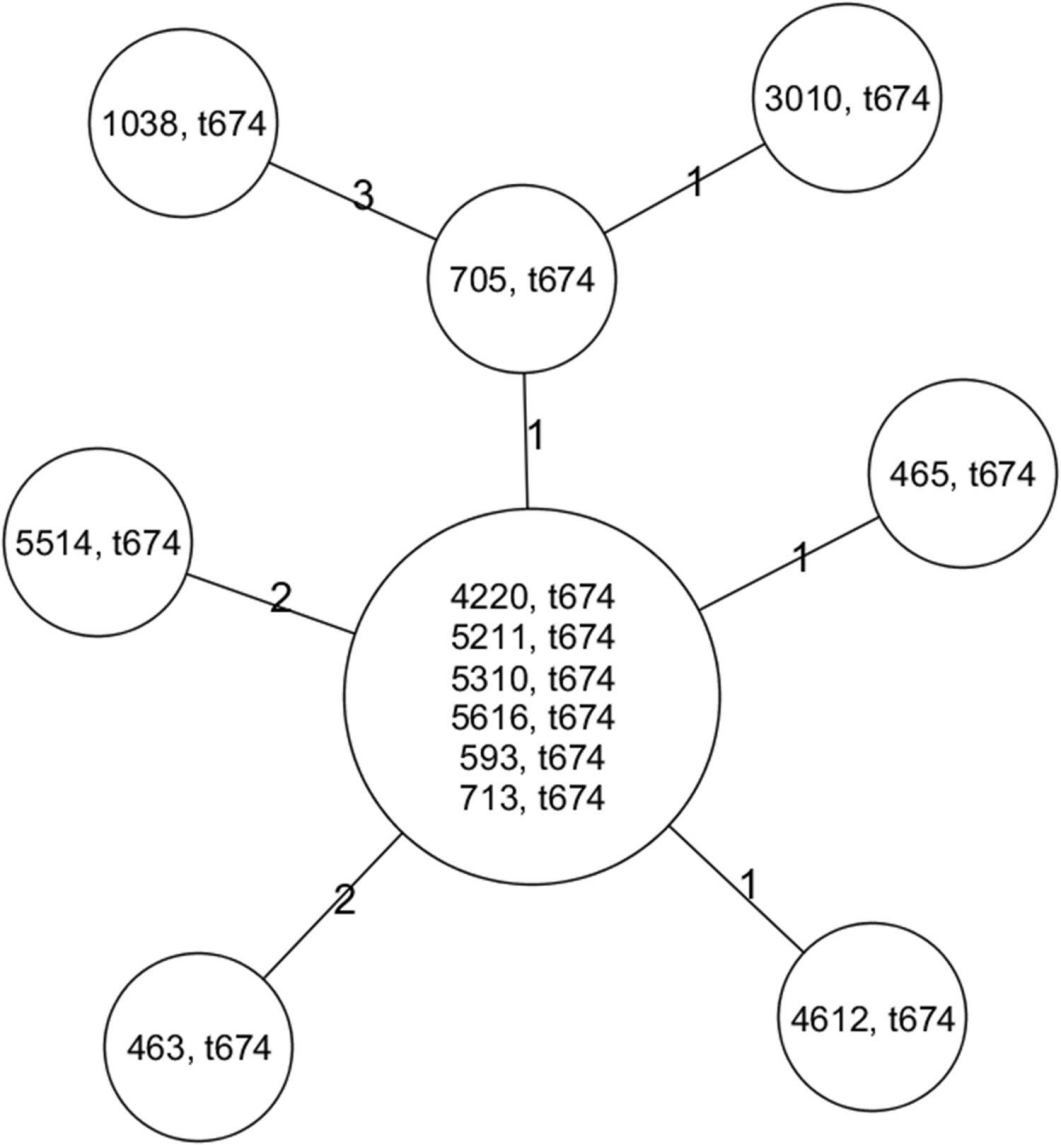
Minimum spanning tree of randomly selected *Staphylococcus aureus* belonging to *spa* type t674 (ST 15). The tree was constructed based on up to the 1,861 genes of the *S. aureus* core genome (cg)MLST. The first numbers in each node indicate the fly identification number while the second number indicates the *spa* type. The numbers on the lines connecting each node indicate the number of differing alleles.

A total of 275 flies (13.8%) from 45 sites were colonized with *S. aureus*. Since *S. schweitzeri* is frequent in African wildlife, we tested if some isolates were misidentified by MALDI-TOF as *S. aureus*. All isolates were *nuc* positive and harboured the 160 bp-fragment of NRPS, thus ruling out *S. schweitzeri* in our collection^9^. The most predominant *spa* type was t674 (98%, 270/275) followed by singular occurrences of t1980, t5305 and t6313 (0.4%, 1/275 each, two isolates were not *spa* typeable). The four distinctive *spa* types belonged to MLST ST15 and showed similar *spa* repeat patterns (t674: 07-34-12-23-02-12-23, t1980: 07-34-12-23, t5305: 07-34-12-23-02-12, t6313: 07-34-12-23-12-23). Since the majority of isolates belonged to t674, WGS was used to increase the discriminatory power in order to distinguish these isolates (Fig. 2). All isolates belonging to t674 differed by ≤ 3 alleles from the most closely related isolate.

The predominant antimicrobial resistance was against clindamycin (8.4%, 23/275) followed by tetracycline (2.2%, 6/275), penicillin (1.5%, 4/275), oxacillin (1.5%, 4/275), rifampicin (1.5%, 4/275), fusidic acid (0.8%, 2/275) and trimethoprim/sulfamethoxazole (0.4%, 1/275). All *S. aureus* isolates were susceptible to fluoroquinolones, macrolides, glycopeptides, gentamicin, linezolid, tigecycline and daptomycin. Four isolates were MRSA-ST15 (*mecA* positive, t5305 [n = 1], t674 [n = 3]). They were found in two different sampling spots (05°56′486" N, 007°25′448" E and 06°26′048" N, 007°28′805" E). All isolates were negative for *lukF*-PV/*lukS*-PV and all penicillin-resistant isolates (1.5%, 4/275) were positive for *blaZ*. *S. aureus* isolates were positive for *chp* (100%, 275/275) and *scn* (97%, 268/275) while none carried *sak* (0%, 0/275). The staphylococcal enterotoxin gene *sei* was predominant (26%, 70/275) followed by *sea* (12%, 32/275). Other staphylococcal enterotoxins *(seb, sec, sed, see, sef, seg* and *seh*) were not detected.

While *S. aureus* positive flies were less likely detected in semi-urban settings, a lower mean temperature, higher air pressure and a lower wind force were environmental factors that favoured the detection of flies with an intestinal colonization of *S. aureus* (Table 1).

The fly species that carried *S. aureus* (n = 275) were *M. domestica *(47.3%, 130/275), followed by *Chrysomya putoria* (20.4%, 56/275), *Musca sorbens* (19.6%, 54/275), *Sacrophaga africa* (8.0%, 22/275), *Chrysomya megacephala* (2.9%, 8/275), *Lucilia cuprina* (1.5%, 4/275) and *M. crassirostris* (0.3%, 1/275, Table S1). The randomly selected non-colonized fly species (n = 109) were *M. domestica* (41.3%, 45/109), *C. putoria* (26.6%, 29/109), *M. sorbens* (13.8%, 15/109), *S. africa* (9.2%, 10/109), *Chrysomya albiceps* (3.7%, 4/109), *Lucilia porphyrina* (2.8%, 3/109), *Sacrophaga cultellata* (1.8%, 2/109) and *Sacrophaga crassipalsi* (0.9%, 1/109). *S. aureus* was neither associated with a specific fly species (Table S1) nor with the genus of the fly (p ≥ 0.08).

### ESBL-E

A total of 16 flies (0.8%) collected from seven sites (6.4%) carried ESBL-E (Fig. 1) and were found mostly within a radius of 2.0 km of the Abia State University Hospital (5° 49′ 278" N, 7° 23′ 772" E). ESBL-producing *E. coli* (n = 15) was predominant followed by *Enterobacter cloacae* complex (n = 1). ESBL-producing *E. coli* were resistant to ceftriaxone (100%, n = 15/15), ceftazidime (93%, n = 14/15), aztreonam (100%, n = 15/15), ciprofloxacin (53%, n = 8/15) and trimethoprim-sulfamethoxazole (100%, n = 15/15). All ESBL-E were susceptible to piperacillin/tazobactam, carbapenems, amikacin, tigecycline, fosfomycin and colistin.

The most frequent ESBL group was *bla*_CTX-M_ (100%, 16/16), followed by *bla*_TEM_ (33%, 8/16) with no occurrence of *bla*_SHV_. CTX-M-15 was the most predominant subgroup (10/16), followed by CTX-M-1 (5/16) and CTX-M-27 (1/16). Among TEM beta-lactamases, TEM 1 was predominant (7/8) followed by TEM 116 (1/8). All ESBL isolates were negative for the *bla*_CMY-2_ genes.

**Figure 3:**
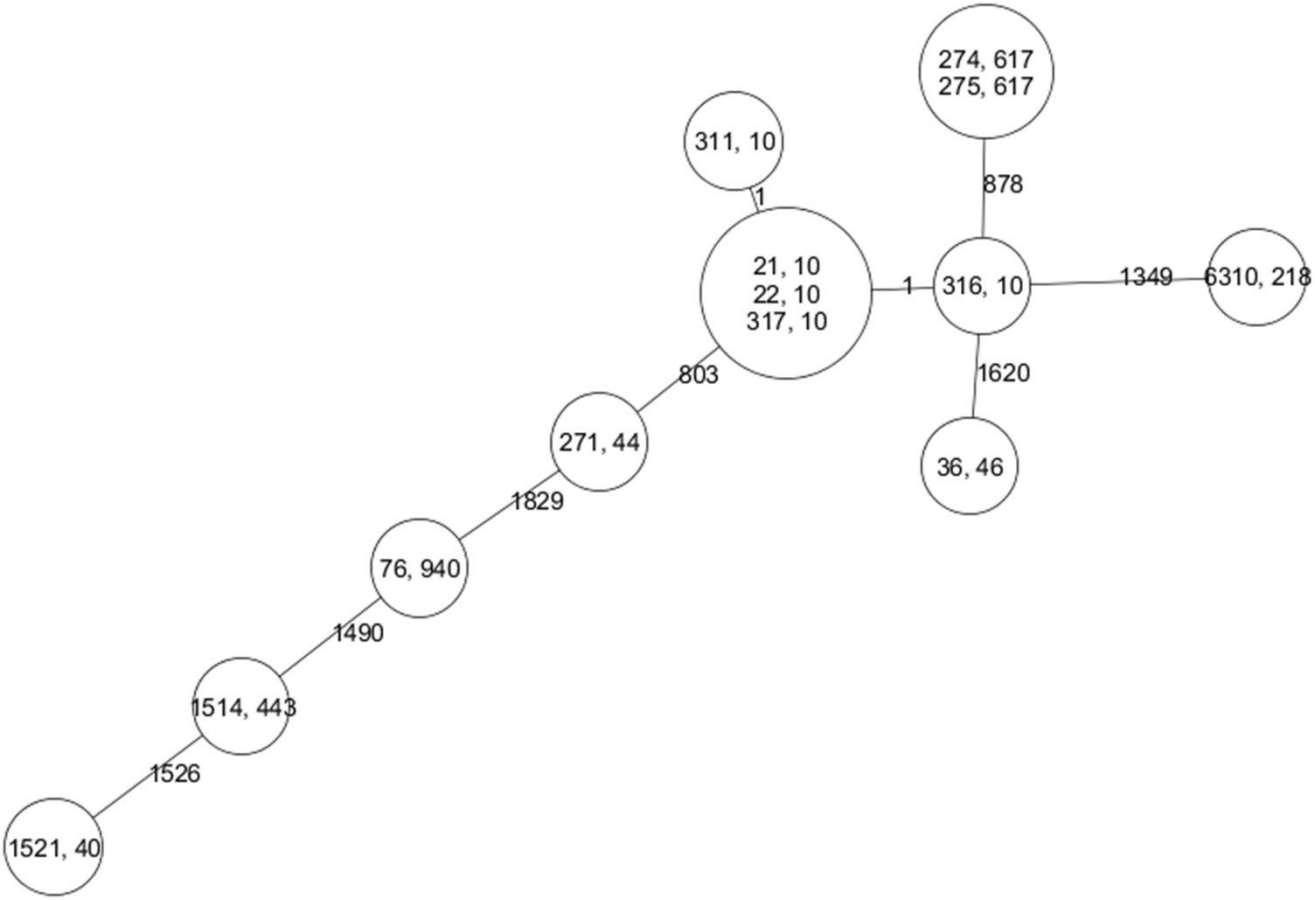
Minimum spanning tree of 13 non-phylogroupable ESBL-producing *Escherichia coli*. The tree was constructed based on the allelic profile of the up to 4671 genes included in the core genome multilocus sequence typing (cgMLST) scheme of the reference strain Sakai. The first number in each circle indicate the identifier of the fly while the second number indicates the MLST sequence type (ST). The numbers on the lines connecting each circle indicates the number of differing alleles.

The majority of ESBL-producing *E. coli* were not grouped in any phylogroup and were consequently subjected to MLST (ST10 [n = 5], ST 617 [n = 2], and singular occurrences of ST40, ST44, ST46, ST218, ST443 and ST940, Fig. 3). Only two isolates belonged to phylogroup D (n = 1) and B2 (n = 1).

The predominant fly species that carried ESBL-E were *M. domestica* (44.0%, 7/16), *M. sorbens* (38.0%, 6/16), *C. putoria* (12.0%, 2/16) and *S. africa* (6.0%, 1/16).

### Enteropathogenic bacteria

None of the flies carried *Salmonella* sp., *Shigella* sp., *Campylobacter* sp. or *Yersinia* sp.

## Discussion

A total of 2,000 flies from Southern Nigeria were screened for the intestinal colonization with antimicrobial resistant and enteropathogenic bacteria. Our main findings are a high proportion of *S. aureus* (13.8%, 275/2,000) and low occurrence of ESBL-E (0.8%, 16/2,000).

The analysis of the intestinal culturome was done to assess the occurrence of bacterial species independent of the resistance phenotype. In general, *Enterobacterales* were only rarely reported (e.g. *K. pneumoniae*, n = 1/82, Figure S1) which is in line with an overall low colonization rate with ESBL-E in flies (0.8%). The vast majority of isolates belonged to the genera *Bacillus* and *Clostridioides* suggesting a selection of spore-forming bacteria by the treatment with ethanol. However, we confirmed the observation that ethanol sanitation of the exoskeleton does not alter the intestinal colonization^10^. Although the culturome was assessed under aerobic conditions, some *Clostridioides* species were detected. This applies for those species, that are known to be aerotolerant (i.e. *C. histolyticum*, *C. terium*, Fig. S1)^11^.

The *S. aureus* intestinal colonization rate in our study (13.8%) is in stark contrast to a 0.4% colonization rate in flies in a comparable approach (i.e. same trap and bait) from Germany^12^. The differing proportions are most likely due to the different settings (tropical vs. temperate regions). All *S. aureus* from our study shared a very similar genetic background based on the *spa* repeat pattern, ST and cgMLST (Fig. 2). This is suggestive either for a common source for all isolates from flies or a cross-contamination between flies during sampling or an adaptation (due to e.g. fitness factors) of this ST15 lineage to the intestinal tract of the flies. An artificial contamination of flies would challenge the scientific significance of our work. However, the widespread detection of *S. aureus* from various sampling site (Fig. 1), both the absence and presence of *mecA* in isolates belonging to t674, different antimicrobial resistance rates, the high diversity of ESBL-E sampled simultaneously and the known effective sanitation of the exoskeleton by ethanol argues against a cross-contamination during sampling^13^. All isolates belonged to ST15, which is in line with a predominance of the clonal complex CC15 in isolates from community-acquired infection in sub-Saharan Africa^14^. In addition, CC15 is known to be well adapted to the human host^15^. However, the low antimicrobial resistance rates, the absence of *lukF*-PV/*lukS*-PV and *sak* might also suggest that *S. aureus* from flies are rather of animal (e.g. livestock or wildlife) than of human origin^16–18^. However, other factors not investigated in our study (e.g. adaptation of *S. aureus* to the gut of ‘filth flies’, fitness factors) could explain the predominance of ST15/t674-*S. aureus* in flies.

Among all *S. aureus* from ‘filth flies’, we only detected *sea* and *sei*. The superantigenic activity of both is superior to other staphylococcal enterotoxins (e.g. members of the SEB group) due to an additional high-affinity MHC II binding site^19^.

The intestinal colonization rate of ‘filth flies’ with ESBL-E was low (0.8%, 16/2,000) compared to a similar study from Germany (3.3%, 44/1346)^12^. This finding is surprising as ESBL-E rates are high both in asymptomatic carriers (33.6%, rectal) and among *Enterobacterales* from bloodstream infection (12.1–15%) in sub-Saharan Africa^20–22^.

Although our study has strengths (e.g. large sample size, broad bacterial culture), some limitations need to be addressed: first, culturome results revealed the abundance of *Bacillus* and *Clostridioides* suggesting a selection of spore-forming genera by ethanol. This might point towards a methodological bias of our work. However, others have also reported a higher prevalence of *Bacillus cereus* than *Enterobacteriaceae*^23^. Second, despite poor sanitation systems and access of flies to human and animal faeces, we did not detect any other enteropathogens (e.g. *Salmonella* sp., *Shigella* sp.). Since we were unable to culture the fly samples immediately, some isolates might have not stayed viable during storage and transport.

In conclusion, diptera-borne *S. aureus* food poisoning might be or become a health issue in the study region due to the high prevalence of enterotoxins (*sea*, *sei*) in *S. aureus* from ‘filth flies’. In contrast, a transmission of ESBL-E through flies by defecation and regurgitation does not seem to play a major role.

## Materials and methods

### Ethical statement

An ethical approval is not required for the analysis of invertebrates. All methods were carried out in accordance with relevant guidelines and regulations. The local Ph.D. committee, Medical Faculty, Westfälische Wilhelms-Universität Münster, approved all experimental protocols.

### Study area/mapping

‘Filth flies’ were collected in Southern Nigeria between June and July 2017. Sampling sites were classified into “urban”, “semi-urban” and “rural” according to the European Union Methodological manual on territorial typologies^24^. GPS coordinates were taken for each sampling site (eTrex 10, Garmin, Olathe, Kansas, USA). For every sampling spot, key environmental conditions were documented (i.e. livestock/human faeces within 10 m radius, presence of refuse dump, presence of decomposing organic matters and setting [urban, semi-urban, rural]). Atmospheric conditions (i.e. humidity, temperature, air pressure, wind force, sunshine hours) were recorded as reported by the Nigerian Meteorological Agency (NiMet, https://nimet.gov.ng/).

The maps were downloaded as jpg-files from openstreetmap (https://www.openstreetmap.org) with bounding boxes (latitude, longitude) related to the region of interest and used as a background for the plots. All plots were produced with “R” (Version 3.6.2) using the package “ggplot2” (grammar of graphics, version 3.2.0)^25^. The data used for the plots were taken from the original files and, where necessary, transformed by methods from the package “dplyr” (grammar of data manipulation, version 0.8.3).

### Diptera

Flies (n = 2,000) were collected using the Gaze trap method^12^. Insect bait, conventionally made from animal proteins, carbohydrates and sugar (Feldner, Waldsee, Germany) in a container covered with gaze and placed under the gaze trap was used to lure the flies. Trapped flies (approximately 20/sampling site) were collected and killed in 70% ethanol and dried in silica gel (2–4 mm, Carl Roth, Karlsruhe, Germany). Ethanol sanitizes the outer surfaces of the flies, avoiding cross-contamination during sampling without altering the intestinal microbiome of the flies^10^.

Each fly was sent for further analysis to Germany in 1 ml sterile sodium chloride (0.45%) at -18 °C (Peli Biothermal Credo Cube, UK).

### Culturome of random flies

We analysed the culturome of 82 randomly selected flies in order to describe the overall colonization pattern of flies with bacteria independent of the AMR phenotype. Since our target bacterial species (e.g. *Escherichia coli*, *Klebsiella* sp.) are aerobic bacteria, we did not use specific anaerobic conditions for culture. After removal of the legs and wings for molecular species identification, the remaining body (head, thorax and abdomen) was mechanically homogenized and cultured in 1 ml BHI broth overnight (37 °C, ambient air). A total of 10 µl of overnight culture was sub-cultured on MacConkey Agar (Oxoid GmbH, Wesel, Deutschland), Columbia Blood Agar (Oxoid), Trypticase Soy Agar (BD, Heidelberg, Germany), Kimmig Agar (Oxoid), Chocolate Agar (BD) and Colistin-Aztreonam (CAP) Agar (Oxoid). All phenotypically different colonies were selected for species identification using Matrix-Assisted Laser Desorption/Ionization-Time-Of-Flight (MALDI-TOF, microflex LT, Bruker Daltonics, Bremen, Germany).

### ESBL-E and enteropathogenic bacteria

After mechanical homogenization, flies (i.e. head, thorax and abdomen) were cultured in 1 ml BHI overnight (37 °C, ambient air). Of this overnight culture, 10 µl were sub-cultured each on Columbia Blood Agar (for growth control) and selective media for the detection of *S. aureus* (SAID, bioMérieux, Marcy l’Etoile, France), ESBL-E (chromID, bioMérieux), *Shigella* sp. and *Salmonella* sp. (SS agar, Oxoid), *Campylobacter* sp. (Campylobacter, Oxoid) and *Yersinia* sp. (CIN, Oxoid). For *Salmonella* sp., 10 µl of the overnight culture was additionally transferred to Müller-Kaufmann Tetrathionate-Novobiocin broth (Oxoid) for a second overnight enrichment step and then sub-cultured on Önöz Agar (Oxoid).

### Identification and characterization

Species of suspected *S. aureus* was identified using MALDI-TOF and confirmed by the detection of a species specific polymorphism of the non-ribosomal peptide synthetase (NRPS)^26^ and the *S. aureus* specific thermostable nuclease (*nuc*)^27^. All *S. aureus* were screened for the immune evasion cluster (IEC)^28^, the Panton-Valentine leucocidin gene (*lukS*-PV/*lukF*-PV)^29^ and the enterotoxin genes *sea*, *seb*, *sec*, *sed*, *see*, *sef*, *seg*, *seh* and *sei*^30,31^.

*Enterobacterales* were identified with VITEK2 automated systems (bioMérieux) due to ambiguous delineation of *E. coli* and *Shigella* sp. using MALDI-TOF.

### Antimicrobial resistance

The antimicrobial susceptibility testing was done with VITEK2 automated systems (bioMérieux) using EUCAST clinical breakpoints (Version 9.0). ESBL-E were confirmed using the double disc diffusion test (Mast diagnostics, Bootle, UK) and were screened for the presence of *bla*_SHV_, *bla*_CTX-M_, *bla*_TEM_ and *bla*_CMY-2_^32,33^. Subtypes of the detected beta-lactamase genes were determined by Sanger sequencing.

All *S. aureus* isolates were screened for the staphylococcal beta-lactamase *blaZ*^34^.

### Genotyping

All *S. aureus* were *spa* typed and one isolate per *spa* type was selected for multilocus sequence typing (MLST)^35,36^. Using an online randomization tool (www.random.org), 13 *S. aureus* isolates were randomly selected for whole genome sequencing (WGS). This was done in order to understand the extent of similarity between the isolates on a whole genome level.

ESBL*-*producing *E. coli* were phylogrouped^37^. Non-phylogroupable strains were subjected to WGS to deduce the MLST sequence type (ST).

### Whole genome sequencing

Genomes of selected isolates of ESBL-E and *S. aureus* were sequenced on an Illumina MiSeq platform (Illumina Inc., San Diego, USA) with a ≥ 75-fold coverage. Quality trimming and de novo assembly using the Velvet assembler was done with SeqSphere + (version 5.9.0; Ridom GmbH, Münster, Germany)^38^. Neighbor-joining trees were constructed based on the cgMLST scheme to assess the clonal relation of the tested isolates^39,40^.

### Fly species identification

All flies being colonized with the target organisms (n = 291) plus 109 randomly selected non-colonized flies from each of the sampling spots were selected for species identification. Legs and wings of flies were crushed using a sterile pestle for DNA extraction (QIAamp DNA Mini Kit, Qiagen, Hilden, Germany). The washing and DNA purification were done according to the manufacturer’s instruction. After amplification, the cytochrome oxidase gene (*coi*) was Sanger sequenced and query sequences were aligned with sequences deposited at the BLAST database for nucleotides^41^. The best match (≥ 99% coverage, ≥ 99% identity) was selected for species identification.

### Statistical analysis

Proportions of categorical variables (e.g. colonization with ESBL-E, *S. aureus*) were compared using the Pearson's chi-squared test or Fisher’s exact test where appropriate and normally distributed continuous variables were compared with student’s t-test. All environmental factors potentially associated with the detection of *S. aureus* in flies (*p* < 0.25) were entered in a multivariate logistic regression model with a stepwise backward elimination^42^. The significance level was set at 0.05. Analyses were done using “R”^25^.

The datasets generated during and/or analysed during the current study as well as all bacterial strains are available from the corresponding author on reasonable request.

## Supplementary information

Supplementary file1

## References

[1] Feyereisen R (2006). Evolution of insect P450. Biochem. Soc. Trans..

[2] Wiegmann BM, Richards S (2018). Genomes of Diptera. Curr. Opin. Insect Sci..

[3] Onwugamba F (2018). The role of `filth flies' in the spread of antimicrobial resistance. Travel Med. Infect. Dis..

[4] Graczyk TK, Knight R, Tamang L (2005). Mechanical transmission of human protozoan parasites by insects. Clin. Microbiol. Rev..

[5] Kobayashi M (1999). Houseflies: not simple mechanical vectors of enterohemorrhagic *Escherichia coli* O157:H7. Am. J. Trop. Med. Hyg..

[6] Madec JY, Haenni M, Nordmann P, Poirel L (2017). Extended-spectrum beta-lactamase/AmpC- and carbapenemase-producing *Enterobacteriaceae* in animals: a threat for humans?. Clin. Microbiol. Infect..

[7] Usui, M., Shirakawa, T., Fukuda, A. & Tamura, Y. The Role of Flies in Disseminating Plasmids with Antimicrobial-Resistance Genes Between Farms. *Microbial drug resistance (Larchmont, N.Y.)* **21**, 562–569. 10.1089/mdr.2015.0033 (2015). 10.1089/mdr.2015.0033 26061440

[8] Odetoyin B, Adeola B, Olaniran O (2020). Resistance patterns of bacterial species isolated from the body surface of the housefly (*Musca domestica*) in Akure, Ondo State, Nigeria. J Arthropod Borne Dis..

[9] Becker, K. *et al.* Implications of identifying the recently defined members of the *Staphylococcus aureus* complex *S. argenteus* and *S. schweitzeri*: a position paper of members of the ESCMID Study Group for Staphylococci and Staphylococcal Diseases (ESGS). *Clin. Microbiol. Infect.*10.1016/j.cmi.2019.02.028 (2019). 10.1016/j.cmi.2019.02.028 30872103

[10] Gupta, A. K. *et al.*Phylogenetic characterization of bacteria in the gut of house flies (*Musca domestica L*.). *FEMS Microbiol. Ecol.* **79**, 581–593. 10.1111/j.1574-6941.2011.01248.x (2012). 10.1111/j.1574-6941.2011.01248.x 22092755

[11] Wells, C. L. & Wilkins, T. D. in *Medical Microbiology* (ed S. Baron) (The University of Texas Medical Branch at Galveston, 1996). 21413252

[12] Schaumburg F (2016). A geospatial analysis of flies and the spread of antimicrobial resistant bacteria. Int. J. Med. Microbiol..

[13] Sproston EL (2010). Multi-locus sequence types of *Campylobacter* carried by flies and slugs acquired from local ruminant faeces. J. Appl. Microbiol..

[14] Ruffing U (2017). Community-associated *Staphylococcus aureus* from Sub-Saharan Africa and Germany: a cross-sectional geographic correlation study. Sci. Rep..

[15] Richardson EJ (2018). Gene exchange drives the ecological success of a multi-host bacterial pathogen. Nat. Ecol. Evol..

[16] Olatimehin A (2018). *Staphylococcus aureus* Complex in the Straw-Colored Fruit Bat (*Eidolon helvum*) in Nigeria. Front. Microbiol..

[17] Schaumburg F (2012). Highly divergent *Staphylococcus aureus* isolates from African non-human primates. Environ. Microbiol. Rep..

[18] Senghore M (2016). Whole-genome sequencing reveals transmission of *Staphylococcus aureus* from humans to green monkeys in The Gambia. Appl. Environ. Microbiol..

[19] Fisher EL, Otto M, Cheung GYC (2018). Basis of virulence in enterotoxin-mediated staphylococcal food poisoning. Front. Microbiol..

[20] Schaumburg F (2013). High burden of extended-spectrum beta-lactamase-producing *Enterobacteriaceae* in Gabon. J. Antimicrob. Chemother..

[21] Flokas ME, Karanika S, Alevizakos M, Mylonakis E (2017). Prevalence of ESBL-producing *Enterobacteriaceae* in pediatric bloodstream infections: a systematic review and meta-analysis. PLoS ONE.

[22] Toy T (2019). Multicountry distribution and characterization of extended-spectrum beta-lactamase-associated gram-negative bacteria from bloodstream infections in Sub-Saharan Africa. Clin. Infect. Dis..

[23] Bahrndorff, S., de Jonge, N., Skovgard, H. & Nielsen, J. L. Bacterial communities associated with houseflies (*Musca domestica* L.) sampled within and between farms. *PLoS ONE* **12**, e0169753. 10.1371/journal.pone.0169753 (2017). 10.1371/journal.pone.0169753 PMC5232358 28081167

[24] Regional statistics team. *Updated urban-rural typology: integration of NUTS 2010 and the latest population grid*, <https://ec.europa.eu/eurostat/statistics-explained/index.php?title=Archive:Urban-rural_typology_update&oldid=209496> (2013).

[25] R: A Language and Environment for Statistical Computing (R Foundation for Statistical Computing, 2019).

[26] Zhang DF (2016). Identification of *Staphylococcus argenteus* in Eastern China based on a nonribosomal peptide synthetase (NRPS) gene. Fut. Microbiol..

[27] Brakstad OG, Aasbakk K, Maeland JA (1992). Detection of *Staphylococcus aureus* by polymerase chain reaction amplification of the nuc gene. J. Clin. Microbiol..

[28] van Wamel WJB, Rooijakkers SHM, Ruyken M, van Kessel KPM, van Strijp JAG (2006). The innate immune modulators staphylococcal complement inhibitor and chemotaxis inhibitory protein of *Staphylococcus aureus* are located on β-hemolysin-converting bacteriophages. J. Bacteriol..

[29] Lina, G. *et al.* Involvement of panton-valentine leukocidin-producing staphylococcus aureus in primary skin infections and pneumonia. *Clin. Infect. Dis.*10.1086/313461 (1999). 10.1086/313461 10524952

[30] Becker K (2003). Prevalence of genes encoding pyrogenic toxin superantigens and exfoliative toxins among strains of *Staphylococcus aureus* isolated from blood and nasal specimens. J. Clin. Microbiol..

[31] Becker K, Roth R, Peters G (1998). Rapid and specific detection of toxigenic *Staphylococcus aureus*: use of two multiplex PCR enzyme immunoassays for amplification and hybridization of staphylococcal enterotoxin genes, exfoliative toxin genes, and toxic shock syndrome toxin 1 gene. J. Clin. Microbiol..

[32] Monstein HJ (2007). Multiplex PCR amplification assay for the detection of *bla*_SHV_, *bla*_TEM_ and *bla*_CTX-M_ genes in *Enterobacteriaceae*. APMIS.

[33] Souna D, Amir AS, Bekhoucha SN, Berrazeg M, Drissi M (2014). Molecular typing and characterization of TEM, SHV, CTX-M, and CMY-2 beta-lactamases in *Enterobacter cloacae* strains isolated in patients and their hospital environment in the west of Algeria. Med. Mal. Infect..

[34] Kaase M (2008). Comparison of phenotypic methods for penicillinase detection in *Staphylococcus aureus*. Clin. Microbiol. Infect..

[35] Enright MC, Day NPJ, Davies CE, Peacock SJ, Spratt BG (2000). Multilocus sequence typing for characterization of methicillin resistant and methicillin susceptible clones of *Staphylococcus aureus*. J. Clin. Microbiol..

[36] Mellmann, A. *et al.* Automated DNA sequence-based early warning system for the detection of methicillin-resistant Staphylococcus aureus outbreaks. *PLoS Med.*10.1371/journal.pmed.0030033 (2006). 10.1371/journal.pmed.0030033 PMC1325475 16396609

[37] Clermont O, Christenson JK, Denamur E, Gordon DM (2013). The Clermont *Escherichia coli* phylo-typing method revisited: improvement of specificity and detection of new phylo-groups. Environ. Microbiol. Rep..

[38] Mellmann A (2016). Real-time genome sequencing of resistant bacteria provides precision infection control in an institutional setting. J. Clin. Microbiol..

[39] Leopold SR, Goering RV, Witten A, Harmsen D, Mellmann A (2014). Bacterial whole-genome sequencing revisited: portable, scalable, and standardized analysis for typing and detection of virulence and antibiotic resistance genes. J. Clin. Microbiol..

[40] Mellmann A (2017). High interlaboratory reproducibility and accuracy of next-generation-sequencing-based bacterial genotyping in a ring trial. J. Clin. Microbiol..

[41] Zehner R (2004). Genetic identification of forensically important flesh flies (Diptera: Sarcophagidae). Int. J. Legal Med..

[42] Schaumburg F (2019). Acquisition and colonization dynamics of antimicrobial-resistant bacteria during international travel: a prospective cohort study. Clin. Microbiol. Infect..

