# Supplementary material for: Antimicrobial resistant and enteropathogenic bacteria in ‘filth flies’: a cross-sectional study from Nigeria
Source: Sci Rep. 2020 Oct 12;10:16990. doi: 10.1038/s41598-020-74112-x (PMC7552403; doi:10.1038/s41598-020-74112-x)
Supplement: Supplementary file 1 — Supplementary file1 [file 41598_2020_74112_MOESM1_ESM.docx]

**Supplement**

**Title:**

Antimicrobial resistant and enteropathogenic bacteria in ‘filth flies’: A cross-sectional study from Nigeria

**Authors:**

Francis Chinedum Onwugamba^1^, Alexander Mellmann^1,2^, Victor Oluoha Nwaugo^3^, Benno Süselbeck^4^, Frieder Schaumburg^1, *^

**Affiliations:**

^1^Institute of Medical Microbiology, University Hospital Münster, Domagkstr. 10, 48149 Münster, Germany

^2^Institute for Hygiene, University Hospital Münster, Robert-Koch 41, 48149 Münster, Germany

^3^Abia State University, PMB 2000 Uturu, Abia State, Nigeria

^4^Center for Information Processing, University of Münster, Röntgenstraße 9–13, 48149 Münster, Germany

*Corresponding author: [frieder.schaumburg@ukmuenster.de](mailto:frieder.schaumburg@ukmuenster.de)

**
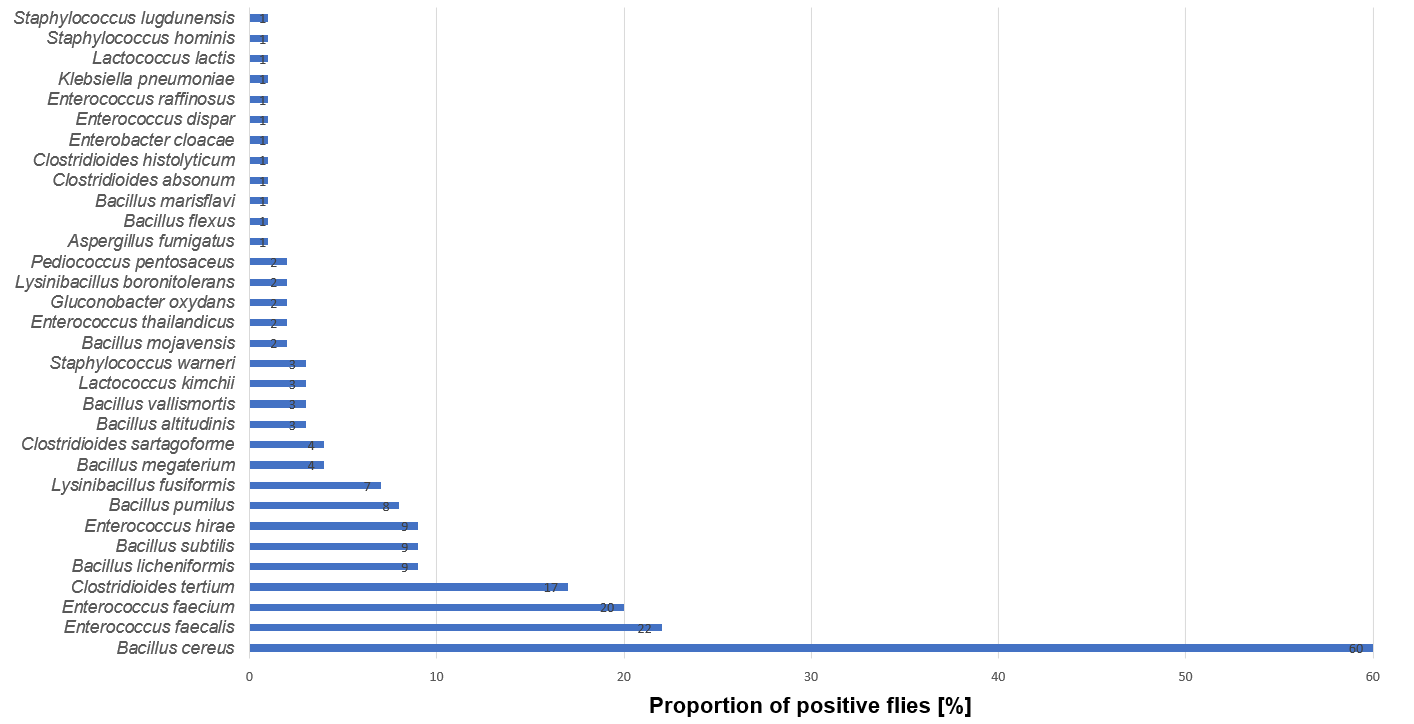
**

**Figure S1:** Intestinal bacterial colonization of randomly selected flies. Flies (n=82) were cultured on several selective and non-selective media under aerobic conditions to assess the background bacterial composition (e.g. prevalence of *Enterobacterales* independent from ESBL phenotype) after processing the flies.

**Table S1:** Distribution of *Staphylococcus aureus* among fly species

| Fly species | *S. aureus* positive  (n=275) | *S. aureus* negative (n=109) | OR (95% CI) | p-value |
| --- | --- | --- | --- | --- |
| *Musca domestica* | 130 (47.3%) | 45 (41.3%) | 1.5 (0.9–2.6) | 0.2 |
| *Chrysomya putoria* | 56 (20.4%) | 29 (26.6%) | Reference |  |
| *Musca sorbens* | 54 (19.6%) | 15 (13.8%) | 1.9 (0.9–3.9) | 0.09 |
| *Sacrophaga africa* | 22 (8%) | 10 (9.2%) | 0.9 (0.4–2.0) | 0.8 |
| Others | 13 (5%) | 10 (9.2%) | 0.9 (0.4–2.1) | 0.8 |

Note: All values are n (%)
